# Validity and reliability of the Foot Function Index (FFI) questionnaire Brazilian-Portuguese version

**DOI:** 10.1186/s40064-016-3507-4

**Published:** 2016-10-18

**Authors:** Bruna Reclusa Martinez, Isabela Maschk Staboli, Danilo Harudy Kamonseki, Elly Budiman-Mak, Liu Chiao Yi

**Affiliations:** 1 Department of Human Movement Sciences, Federal University of São Paulo, Rua Silva Jardim, 136, Santos, SP CEP 11015-020 Brazil; 2 U.S. Department of Veterans Affairs, Washington, DC USA

**Keywords:** Foot diseases, Questionnaires, Outcome assessment, Validation studies

## Abstract

**Purpose:**

To evaluate the validity and reliability of the Foot Function Index (FFI) in its Brazilian Portuguese version.

**Methods:**

The validity and reliability of the FFI were tested in 50 volunteers, with plantar fasciitis, metatarsalgia and chronic ankle sprain. The FFI validity process used the Short Form-36 (SF-36) and Foot and Ankle Outcome Score (FAOS) questionnaires. The correlation between FFI, SF-36 and FAOS was done using the Pearson’s linear coefficient. The inter and intra-evaluator reliability was ascertained by means of the intraclass correlation coefficient (ICC) and the internal consistency by means of Cronbach’s alpha coefficient. The scores were used to assess the standard error measurement (SEM), minimal detectable change (MDC) and ceiling floor and effects.

**Results:**

The validity process showed that there were correlations between FFI and the “pain” and “social aspects” subscales of SF-36 and all subscales of FAOS, except for “other symptoms”. The Brazilian-Portuguese version of FFI showed excellent intra and interevaluator correlations, with an ICC range of 0.99–0.97 and score reliability that was considered highly satisfactory, with Cronbach’s alpha range of 0.80–0.61. The SEMs for inter and intra-evaluator reliability were 1.32 and 1.08, respectively. The MDC was 2.42 (90 % confidence interval). No ceiling and floor effect were detected.

**Conclusions:**

The Brazilian-Portuguese version of the FFI questionnaire was found to be a valid and reliable instrument for foot function evaluation, and can be used both in scientific settings and in clinical practice.

## Background

Evaluating musculoskeletal disorders is essential in order to determine physical impairments among individuals. However, due to the impact caused by these disorders on the state of health and quality of life, healthcare professionals are increasingly giving emphasis to analyses from the patients’ perspective. Thus, conventional evaluations such as strength and range of motion are being added through valid measurements that can determine functional, social and emotional characteristics (Eechaute et al. 2007; Hale and Hertel 2005; Hunt and Hurwit 2013; Kirkley and Griffin 2003; Shultz et al. 2013).

One in every five middle-aged person presents foot and ankle pain, and this may compromise locomotion, impairment of balance and a limitation in functional activities of daily living (Thomas et al. 2011). Pathological conditions of the ankle and foot results from therapeutic interventions are under evaluation by healthcare professionals and researchers using self-reported outcome instruments. These instruments make it possible to use of reliable measurements for patients’ perceptions, and specific instruments have been standardized in order to follow up and evaluate the effects of a given intervention (Martin and Irrgang 2007).

The Foot Function Index (FFI) questionnaire (Budiman-Mak et al. 1991) and other four specific instruments for the ankle and foot (Bennett et al.^1998^; Binkley et al.^1999^; Martin et al. 2005; Williams et al. 2003) are the only ones for these body parts that have been identified as presenting positive evidences about their usage. This can be stated because these questionnaires encompass the four categories of content validity, construct validity, reliability and responsiveness (Martin and Irrgang 2007). The FFI has also shown excellent responsiveness through presenting positive level three ratings in relation to its capacity in order to determine changes resulting from an intervention among patients with rheumatoid arthritis (van der Leeden et al. 2008). Additionally, it was classified as the fourth most used scale for orthopedic ankle and foot evaluations over a nine-year period (2002–2011) (Hunt and Hurwit 2013; Martin et al. 2014).

The FFI questionnaire was developed to measure the impact of foot pathologies on function in terms of pain, disability and activity restriction. The questionnaire is composed by 23 items distributed into three subscales: disability (5 items), activity limitation (9 items) and pain (9 items) (Budiman-Mak et al. 1991). It was developed in English (Budiman-Mak et al. 1991) and valid translated versions have been produced for use in Chinese (Wu et al. 2008), German (Naal et al. 2008), French (Pourtier-Piotte et al. 2015), Italian (Martinelli et al. 2014) and Spanish (Paez-Moguer et al. 2014). Because of the importance of standardization when using measures of evaluation, questionnaires developed in foreign languages must be translated and their psychometric properties evaluated, to create equivalence between studies. The FFI questionnaire was recently translated and culturally adapted into Brazilian Portuguese (Yi et al. 2015), and the aim of the present study was to evaluate the validation and reliability of the Brazilian version of the FFI questionnaire.

## Methods

### Subjects

The sample size was calculated by using the total score of the original FFI questionnaire (Budiman-Mak et al. 1991), with 95 % of confidence and 90 % of power and was obtained a number of 46 subjects (Sousa and Rojjanasrirat 2011). To prevent loss of volunteers, 50 patients were enrolled in this study. They were clinically diagnosed with plantar fasciitis (56 %), metatarsalgia (14 %) and lateral sprain of the ankle (30 %) in which the injuries occurred more than 6 months earlier. All these patients were seen at our institution’s Physiotherapy Unit and Orthopedics and Traumatology Outpatient Clinic.

The inclusion criteria for the plantar fasciitis were: (1) plantar medial heel pain, most noticeable with initial steps after a period of inactivity; (2) heel pain precipitated by a recent increase in weight-bearing activity; (3) pain with palpation of the proximal insertion of the plantar fascia; (4) positive windlass test (Martin et al. 2014). The criteria for metatarsalgia were: (1) pain on metatarsals heads during palpation; (2) Mulder positive sign (Espinosa et al. 2008). The lateral ankle sprain criteria were: (1) history of at least 1 significant ankle sprain; (2) inflammatory symptoms including pain and swelling; (3) interruption of the physical activity for at least 1 day after the sprain (Gribble et al. 2014).

The volunteers included in the study were of both genders (76 % female), with an average age of 32.9 ± 14.13 years (range 18–60 years) and all of them attended school until at least fully grading high school. Patients with cognitive alterations, neurological conditions and other lower-limb diseases were excluded.

### Testing the psychometric properties

#### Validity

The validity of the Brazilian version of the FFI questionnaire was tested in order to establish the correlation among the proposed instrument and instruments that had already been translated and validated (Andresen 2000). The Brazilian-Portuguese version of the Medical Outcomes Study 36-item Short Form Health Survey (SF-36) (Ciconelli et al. 1999) and the Foot and Ankle Outcome Score (FAOS) (Imoto et al. 2009) questionnaires were used for the validity process. The questionnaires were applied by the same evaluator, through interviews, as the other questionnaires validity process conducted in Brazil (Ciconelli et al. 1999; Imoto et al. 2009; Yi et al. 2015). The completion time of the FFI questionnaire lasted from 5 to 10 min.

##### FFI score

The calculus to obtain the score for each subscale is done by using the following formula: the sum of the score from all items answered by the patient/divided by the total score possible for the subscale multiplied by 100. If the patient does not perform an activity indicated by one of the subscale items (for example, not using auxiliary devices), this item is considered to be not applicable. Thus, the scores for such items will not be considered in the total sum for the subscale (Budiman-Mak et al. 1991).

The final score of the questionnaire is calculated using the formula: sum of the final percentages of all subscales divided by three (total number of subscales). The results may vary from 0 to 100 % and are directly proportional to the limb’s functional impairment, such that the higher the percentage is, the greater the functional alteration presented by the patient is (Budiman-Mak et al. 1991).

##### Short Form Health Survey (SF-36)

SF-36 is a multidimensional instrument used to evaluate quality of life. It is composed by 36 items distributed into eight scales: physical functioning, physical role functioning, bodily pain, general health perceptions, vitality, social role functioning, emotional role functioning and mental health. The total score from each scale varies from 0 to 100 points, where zero represents the worst state of health and 100 the best state (Ciconelli et al. 1999).

##### Foot and Ankle Outcome Score (FAOS)

FAOS is a questionnaire that is used to evaluate symptoms and functional limitations caused by ankle and foot alterations. The questionnaire consists of 42 items distributed into five subscales: pain (9 items), symptoms (7 items), function in daily living (17 items), function in sports and recreation (5 items) and quality of life (4 items) (Imoto et al. 2009).

The score is calculated for each subscale using the following formula: 100 − (subject score multiplied by 100 divided by the maximum score possible in the subscale). Scores vary from zero to 100, where zero indicates extreme symptoms and 100 indicates absence of symptoms (Imoto et al. 2009).

#### Reliability

Reliability was tested by evaluating the reproducibility (test/retest) and internal consistency (IC). Reproducibility is the extent to which the same results from the questionnaire are obtained through different administrations. Internal consistency indicates the extent to which the items in a subscale are correlated, in order to evaluate homogeneity (Andresen 2000; Eechaute et al. 2007; Kirkley and Griffin 2003).

Inter-observer reliability is measured through applying the FFI questionnaire on two occasions: initially by evaluator A and after a period of at least an hour, by evaluator B, as the other validity questionnaires process conducted in Brazil (Ciconelli et al. 1999; Imoto et al. 2009; Yi et al. 2015). A third administration of the questionnaire was made in order to evaluate intra-observer reliability, performed by evaluator A after a time interval of a maximum of seven days after the first application (Terwee et al. 2007). The questions and alternatives of the FFI were read out loudly to all volunteers, without providing explanations, with the aim of not influencing the responses. During the period between administrations of the questionnaires, the volunteers were instructed to maintain their daily activities.

#### Ceiling and floor effects

Ceiling and floor effects of FFI were calculated. These effects are calculated in order to evaluate the entire spectrum of a condition’s severity with the items it contains (Eechaute et al. 2007). The ceiling and floor effects occur when the maximum or minimum possible score is achieved by a substantial portion of the sample of participants (Eechaute et al. 2007).

### Ethical considerations

This study was approved by the Research Ethics Committee, under protocol number 226.521, and all the participants signed the free and informed consent statement.

### Statistical analyses

Descriptive statistical analysis on the sample was performed using means and standard deviations (SD). The total FFI score was correlated with the subscales of the SF-36 and FAOS questionnaires, by means of Pearson’s linear correlation coefficient. The alpha error was set at p < 0.05.

To ascertain the intra and inter-observer reliability, the interclass correlation coefficient (ICC) was calculated. The IC of the FFI subscales was evaluated by means of Cronbach’s alpha coefficient. The reliability results were combined with the total FFI scores and their subscales in order to define the standard error of measurement (SEM) (Beaton et al. 2000). The SEM was calculated using the following formula: SEM = SD1.√(1 − ICC), where SD1 = is the standard deviation of the initial evaluation and ICC = the ICC found in the reproducibility analysis (Beaton et al. 2000; Leggin et al. 2006; Lopes et al. 2008; Martin and Irrgang 2007).

The error associated with applying the FFI over this time interval also defined the minimal detectable change (MDC), i.e. the minimum amount of change necessary for the FFI score (with 90 % confidence) to be considered to be a real change over the time frame of a maximum of one week. The MDC was calculated using the following formula: MDC = 1.65.√2.SEM (Leggin et al. 2006; Lopes et al. 2008; Martin and Irrgang 2007).

A ceiling and floor effects were considered if more than 15 % of the respondents achieved the lowest or highest (0–100) possible score of FFI (Eechaute et al. 2007).

## Results

### Validity

Table 1 presents the scores obtained (mean, standard deviation and variation) for the FFI, SF-36 and FAOS questionnaires.

**Table 1:** FFI, FAOS and SF-36 questionnaires scores

Questionnaires n = 50	Mean (SD)	Variation
FFI Total (0–100)	20.54 (15.17)	0–59.0
Disability	12.34 (12.91)	0–48.1
Activity limitation	18.89 (14.22)	0–58.0
Pain	30.39 (24.94)	0–96.8
FAOS (0–100)		
Pain	75.11 (15.29)	38.8–97.2
Other symptoms	73.86 (12.53)	35.7–100
Function, daily living	87.68 (11.38)	57.3–100
Function, sports and recreational activities	68.50 (18.82)	25.0–100
Quality of life	45.75 (21.48)	6.25–81.2
SF-36 (0–100)		
Physical functioning	80.40 (18.32)	30.0–100
Physical role functioning	64.00 (35.05)	0–100
Bodily pain	49.54 (21.73)	0–100
General health perceptions	61.62 (23.19)	22.0–100
Vitality	56.50 (20.31)	5.0–90.0
Social role functioning	78.25 (20.33)	25.0–100
Emotional role functioning	53.33 (40.19)	0–100
Mental health	66.48 (20.25)	20.0–100

*FFI* Foot Function Index, *FAOS* Foot and Ankle Outcome Score, *SF-36* Short-Form 36, *n* volunteer number, *SD* standard deviation

When correlating the total score from the FFI questionnaire with the subscales of the SF-36 questionnaire, a correlation between the subscales “bodily pain” (r = −0.36; p = 0.010) and “social role functioning” (r = −0.36; p = 0.011) was found. In relation to the FAOS questionnaire, there were correlations between the total FFI score and the subscales “pain” (r = −0.58; p = 0.001), “function, daily living” (r = −0.39; p = 0.006), “function, sports and recreational activities” (r = −0.50; p = 0.001) and “quality of life” (r = −0.37; p = 0.009) (Table 2). Only the subscale “other symptoms” (r = −0.19; p = 0.187) showed no correlation with the FFI questionnaire.

**Table 2:** Correlation among FFI total score and SF-36–FAOS questionnaires subscales

n = 50	Correlation coefficient (r)	*p* value
SF-36		
Physical functioning	−0.24	0.090
Physical role functioning	−0.11	0.465
Bodily pain	−0.36	0.010*
General health perceptions	−0.15	0.283
Vitality	−0.16	0.276
Social role functioning	−0.36	0.011*
Emotional role functioning	−0.28	0.052
Mental health	−0.22	0.131
FAOS		
Pain	−0.58	0.001**
Other symptoms	−0.19	0.187
Function, daily living	−0.39	0.006*
Function, sports and recreational activities	−0.50	0.001**
Quality of life	−0.37	0.009*

*FFI* Foot Function Index, *SF-36* Short-Form 36, *FAOS* Foot and Ankle Outcome Score, *n* volunteer number, *r* Pearson coefficient correlation

* *p* < 0.05; ** *p* = 0.001

### Reliability

The means and standard deviations of the total FFI scores and subscales for each reproducibility evaluation (test/retest) are presented in Table 3.

**Table 3:** Inter and intra-observer reliability of FFI (total score and subscales)

Scores (n = 50)	Mean (SD)		
	1st interview^a^	2nd interview^b^	3rd interview^c^
	(Evaluator A)	(Evaluator B)	(Evaluator A)
FFI Total	20.54 (15.17)	20.57 (15.01)	20.32 (14.91)
Disability	12.34 (12.91)	11.85 (12.67)	11.62 (12.33)
Activity limitation	18.89 (14.22)	18.71 (13.85)	19.28 (14.43)
Pain	30.39 (24.94)	30.57 (25.12)	30.63 (24.50)

*FFI* Foot Function Index, *n* volunteer number, *SD* standard deviation

^ab^Inter-observer evaluation; ^ac^ Intra-observer evaluation

The reliability of the FFI total score and all the subscales were considered highly satisfactory. For Cronbach’s alpha was found α = 0.78 for the FFI total score and the subscales vary from 0.80 to 0.61 during the first questionnaire administration. The Brazilian-Portuguese version of FFI showed excellent intra and inter-observer reliability, with an ICC 0.99–0.97 (Table 4).

**Table 4:** Inter and intra-observer reliability analysis of FFI questionnaire

Scores (n = 50)	*Alpha* de *Cronbach* (IC)	Inter-observer ICC	Intra-observer ICC
FFI total	0.78 (0.68–0.86)	0.99	0.99
Disability	0.80 (0.71–0.87)	0.98	0.97
Activity limitation	0.61 (0.42–0.74)	0.97	0.98
Pain	0.66 (0.50–0.78)	0.99	0.99

*FFI* Foot Function Index, *n* volunteer number, *IC* internal consistency, *ICC* intra-class coefficient correlation

The standard error of measurement (SEM) associated with the total FFI score was 1.32 points for the inter-observer and 1.08 points for the intra-observer evaluation. The minimal detectable change (MDC) was 2.42 points (90 % confidence interval) over the time interval of the intra-observer evaluation (maximum of one week). The SEM for the FFI domains ranged from 1.08 to 2.16, whereas the MDC ranged from 2.42 to 3.43 (Table 5).

**Table 5:** Standard error of measurement (SEM) and minimum detectable change (MDC) from the FFI scores

Scores (n = 50)	Inter-observer^a^	Intra-observer^b^	
	SEM	SEM	MDC (90 % CI)
FFI Total	1.32	1.08	2.42
Disability	1.77	2.15	3.43
Activity limitation	2.16	1.56	2.91
Pain	2.03	1.82	3.15

*FFI* Foot Function Index, *n* volunteer number, *SEM* standard error of measurement, *MDC* minimal detectable change, *CI* confidence interval

^a^SEM of inter-observer; ^b^SEM and MDC of intra-observer after maximum interval of two weeks

### Ceiling and floor effects

When administered the FFI questionnaire in patients with plantar fasciitis, metatarsalgia and ankle lateral sprain, no volunteer reached the maximum or minimum score of FFI questionnaire, therefore there were no ceiling and floor effects.

## Discussion

From the results of this study, the Brazilian-Portuguese version of the Foot Function Index questionnaire has shown to be a valid and reliable instrument among patients with foot disorders.

For the FFI validation analysis the total FFI score was used because of its greater practicality for clinical application, unlike previous studies that used the scores of each subscale (Budiman-Mak et al. 1991; Martinelli et al. 2014; Naal et al. 2008; Paez-Moguer et al. 2014; Pourtier-Piotte et al. 2015; Wu et al. 2008). In this way, the present study found that the FFI correlated with two SF-36 subscales: “bodily pain” and “social role functioning”. It was found that, if the SF-36 quality-of-life questionnaire by itself for the validation process, as seen in previous validation studies (Budiman-Mak et al. 1991; Martinelli et al. 2014; Naal et al. 2008; Pourtier-Piotte et al. 2015; Wu et al. 2008), social and emotional issues, unrelated to orthopedic injuries, could interfere in the findings. Thus, there was a need to add a specific evaluation measurement for the ankle and foot, and this was the first study to use the FAOS (Imoto et al. 2009) in the FFI validation process.

Application of a specific questionnaire that evaluates symptoms and functional limits for the foot and ankle (FAOS) in the FFI validity process made it possible to establish correlations for all the subscales (“pain”, “function in daily living”, “function in sports and recreation” and “quality of life”), except for “symptoms”. This result shows the great importance of FFI validity, thus making this questionnaire a valid instrument for application in clinical practice. The lack of correlation with the “symptoms” subscale may have occurred because of the diversified characteristics of the questions from which it is composed.

In evaluating reliability, the Brazilian-Portuguese version of FFI showed excellent reproducibility intra-observer (ICC = 0.99), thus indicating that the version is as reliable as its original version (ICC = 0.87) (Budiman-Mak et al. 1991). Furthermore, the reliability of the subscales “pain”, “activity limitation” and “disability” showed excellent reproducibility, with ICC of 0.99, 0.98 and 0.97, respectively. The original version of the FFI questionnaire showed values of 0.69, 0.81 and 0.84, respectively.

In addition to assessing intra-observer reliability with a 1-week interval, inter-observer reliability was assessed in the present study, with at least a 1-h interval after the first evaluation. This was the first study on the FFI to perform inter-observer reliability assessment. From the results, it was found that the Brazilian version was reliable, with excellent reproducibility values with ICC of 0.99, 0.99, 0.97 and 0.98 for “total score”, “pain”, “activity limitation” and “disability”.

The IC evaluated by Cronbach’s alpha was considered adequate (α = 0.78) for FFI total score and showed values of 0.66, 0.61 and 0.80 for the subscales “pain”, “activity limitation” and “disability”. These finding indicates that the items of the Brazilian version are homogenous, as the original version, which showed alpha values of 0.95, 0.94, 0.73 and 0.92 for the “FFI total”, “pain”, “activity limitation” and “disability”, respectively (Budiman-Mak et al. 1991).

This was the first study to use the MDC and SEM as reliability measurements in order to analyze the psychometric properties of the FFI. The MDC was 2.42 points (90 % confidence interval) over the time interval of the intra-observer evaluation (maximum of one week) and the SEM relating to the total FFI score was 1.08 points over the intra-observer and 1.32 points over the inter-observer evaluation. These values show that the level of agreement to the Brazilian version of FFI was considered excellent, representing <5 % of the total score of the FFI (0–100) (Wageck et al. 2013). Furthermore, the Brazilian Version of the FFI questionnaire did not show ceiling and floor effects when administered in patients with foot disorders. This finding indicates that, the Brazilian version of FFI points out that there is no limitation to detect the spectrum of relatively mild foot conditions.

We considered as a limitation of the study, the inclusion of patients with different foot disorders. However, after the validity process in individuals with plantar fasciitis, metatarsalgia and lateral ankle sprain, we found an excellent reliability in determining the functionality in different foot disorders. These findings showed that the FFI questionnaire allowing a large number of people to apply it. Another possible limitation was that, we only used the FFI total score data to correlate with SF-36 and FAOS measures. The subscales were not used. However in clinical practice, the use of the total score indicates the real impact of the foot injuries on the functionality (Budiman-Mak et al. 1991).

The Brazilian-Portuguese version of FFI questionnaire was shown to be valid, highly reproducible regarding both inter and intra-observer evaluation and not show ceiling and floor effects in patients with foot disorders. It therefore allows healthcare professionals to objectively evaluate patients’ health at the beginning, middle and end of their patient’s rehabilitation. For future studies, it is recommended that the responsiveness of this questionnaire should be evaluated, in order to determine whether this instrument is capable of detecting clinical changes in patients after a period of time and also, to identify the normative values of the FFI questionnaire in the Brazilian population. These values will contribute to the correct interpretation of the FFI scores according to the age, gender, and anthropometrics data, to allow the understanding of the clinical improvement after treatment in each evaluated group (Schneider and Jurenitsch 2016).

## Conclusion

The Brazilian-Portuguese version of the FFI questionnaire has shown to be a valid and reliable instrument for foot functioning evaluation, and it can be used in scientific and in clinical practice fields.
